# Management of Resistant Hypertension Based on Recommendations from Different Guidelines and the Systolic Blood Pressure Intervention Trial

**DOI:** 10.7759/cureus.5371

**Published:** 2019-08-12

**Authors:** Sandeep Banga, Sudhir Mungee, Avani R Patel, Shantanu Singh, Tinoy J Kizhakekuttu

**Affiliations:** 1 Cardiology, West Virginia University School of Medicine, Morgantown, USA; 2 Cardiology, University of Illinois College of Medicine, Order of St. Francis Medical Centre, Peoria, USA; 3 Internal Medicine, Northern California Kaiser Permanente, Fremont, USA; 4 Pulmonary Medicine, Marshall University School of Medicine, Huntington, USA; 5 Cardiology, University of Illinois College of Medicine at Peoria, Order of St. Francis Medical Centre, Peoria, USA

**Keywords:** resistant hypertension, resistant hypertension management, jnc 8, sprint study

## Abstract

The long-term management of patients with resistant hypertension has been made even more difficult by a “moving target” goal blood pressure (BP). The Seventh Report of the Joint National Committee on Prevention, Detection, Evaluation, and Treatment of High Blood Pressure (JNC 7) guidelines defined resistant hypertension as the failure to achieve goal BP in patients who are adhering to full doses of an appropriate three-drug regimen that includes a diuretic. The Eighth Report of the Joint National Committee on Prevention, Detection, Evaluation, and Treatment of High Blood Pressure (JNC 8) guidelines laid out more lenient target BP goals, without addressing the definition of resistant hypertension directly. The present scenario is a state of confusion, with providers selectively adopting recommendations from different guidelines. The Systolic Blood Pressure Intervention Trial (SPRINT) trial contributed to the confusion with further evidence supporting the strict control of hypertension. In addition, the failure of another trial on renal denervation in the US has essentially put an end to six long years of experimentation with catheter ablation in patients with resistant hypertension. Other therapies are still experimental. Adding a new dimension of medical management, spironolactone has made a comeback in resistant hypertension, with reports of better responsiveness when added to existing anti-hypertensive therapy. The present review discusses the current state and management options for patients with resistant hypertension considering the new evidence. Newer advances in pharmacological and device therapy are also discussed to improve understanding and quality in the management of resistant hypertension.

## Introduction and background

Resistant hypertension has been historically defined as a blood pressure (BP) measurement that exceeds 140/90 millimeters of mercury (mmHg) (130/80 mmHg in patients with diabetes mellitus or renal insufficiency) despite the regular use of three or more antihypertensive drugs of different classes that should include one diuretic at the maximum (or maximum tolerated) dose unchanged for at least one month without interruption as per the Seventh Report of the Joint National Committee on Prevention, Detection, Evaluation, and Treatment of High Blood Pressure (JNC 7) guidelines [1]. Renal insufficiency has been defined as a creatinine level greater than 1.5 milligrams/milliliters (mg/mL) or proteinuria more than 300 mg in 24 hours. The 2008 American Heart Association (AHA) position statement defined resistant hypertension as uncontrolled hypertension despite treatment with at least three antihypertensive drugs or controlled hypertension with at least four drugs [2].

The latest definition according to the 2017 AHA/American College of Cardiology (ACC) taskforce emphasizes strict control with a target BP <130/80 mmHg and has defined resistant hypertension as uncontrolled BP ≥130/80 mm Hg while on optimal doses of at least three antihypertensives with one being a diuretic. Office blood pressures have been defined as BP <130/80 mmHg with the patient requiring more than four anti-hypertensive medications [3]. The Eighth Report of the Joint National Committee on Prevention, Detection, Evaluation, and Treatment of High Blood Pressure (JNC 8) neglected to address resistant hypertension, although resistant hypertension affects up to a quarter of hypertension patients [4].

## Review

Prevalence and prognosis

Reliable estimates of the prevalence of resistant hypertension are not available. However, estimates based on large clinical trials suggest a recent prevalence of 13% unlike the previously reported rate of 20%-30% amongst all hypertensive patients under treatment [1,5-10]. With the new definition having a lower threshold for target blood pressures, the prevalence should be around 17%. The data from population studies suggest that hypertension-related target organ damage, such as stroke, heart failure, myocardial infarction, and renal failure, are directly related to the level of blood pressure (BP) [11]. There are no studies powered to address the issue of prognosis in patients with resistant hypertension.

Resistant hypertension may be caused by the identified factors, as seen in Table 1.

**Table 1:** Factors Potentially Responsible for Resistant Hypertension These factors should be carefully looked into, as these may be responsible for resistant hypertension.

Factors Responsible for Resistant Hypertension
Excess Alcohol Intake
Volume Overload
Drug-Induced Resistant Hypertension
Identifiable Causes of Secondary Hypertension: Medical Renal Disease, Renovascular Disease, Conn's Syndrome, Obstructive Sleep Apnea, Pheochromocytoma, Thyroid Disease, Cushing Syndrome, Coarctation of Aorta, Intracranial Tumors

Pseudo-resistant hypertension

Pseudo-resistant hypertension is a condition where the patient does not have resistant hypertension but will present with uncontrolled BP despite undergoing the appropriate treatment. The following factors may result in this condition [12-16] as seen in Table 2.

**Table 2:** The Potential Causes of Pseudo-resistant Hypertension [12-16]

Causes of Pseudo-Resistant Hypertension
Blood pressure measurement not done by proper guidelines.
White-Coat Hypertension.
Calcified or atherosclerotic arteries as seen in the elderly.
Poor patient adherence to medications.
Inadequate dosing or inappropriate combinations of anti-hypertensives.
Physician’s inertia to increase dose/number of drugs even if targets are not reached.

Evaluation and management

Physicians will be able to effectively manage resistant hypertension using the following step-by-step principles and management.

1. Physicians must have knowledge of and adhere to the most recent guidelines of resistant hypertension management [1,3].

2. They must identify and reverse the causes of pseudo-resistance [1,3].

3. They must identify and reverse the causes of true resistance:

 a) Have prior knowledge that pharmacological agents like non-steroidal anti-inflammatory drugs (NSAIDS) increase BP and, therefore, discontinue or minimize their use [1,3].

 b) Evaluate the amount of alcohol intake in the patient and try to discontinue the intake [1,3].

 c) Decrease salt intake to less than 2-3 grams (gm) per day [1,3].

 d) Assess the degree of abdominal obesity and increase daily physical activity to 40 minutes per day for a minimum of three to four days/week [1,3].

 e) Evaluate renal function and modify treatment if necessary [1,3].

 f) Identify and manage any potential causes of secondary hypertension [1,3].

 4. Use combinations of anti-hypertensive drugs for the definitive management of the condition [1,3].

Non-pharmacological approach

Similar to many conditions, resistant hypertension can be managed with a non-pharmacological approach. This may require certain lifestyle changes done by the patient and an understanding of factors that can worsen the condition.

1. Daily alcohol intake should be restricted to 1 oz in men and 0.5 oz in women or individuals of lower weight classification [1,3].

2. Excessive dietary intake of salt is seen in many patients with resistant hypertension, which leads to an expanded plasma volume as identified in 90% of patients. Taking less than 3 gm/day of salt is associated with the modest reduction in BP largely seen in African Americans and elderly patients [1,3].

3. Discontinue NSAIDS, as they lead to reduced renal prostaglandin production followed by sodium and water retention. They increase BP by 5 mmHg. Stop anabolic steroids, glucocorticoids, nasal decongestants, and amphetamine-like stimulants [1,3].

4. Identify kidney disease if present, both parenchymal and vascular, in order to select proper antihypertensives, particularly diuretics [1,3].

5. Obese patients have insulin resistance and are associated with increased sympathetic activity and obstructive sleep apnea, all of which contribute to resistant hypertension. Weight loss and a reduced-calorie diet are associated with a modest BP reduction in obese hypertensive patients [1,3].

6. Age greater than 65 years is associated with a higher prevalence of resistant hypertension [1,3].

Pharmacological approach

Resistant hypertension patients may require pharmacological treatment for the effective management of their condition. The following approach is recommended.

1. The present rationale in the treatment of resistant hypertension is to ensure that all possible mechanisms responsible for BP elevations are blocked at all levels [1,3].

2. Suboptimal dosing regimens or inappropriate antihypertensive drug combinations are the most common causes of resistant hypertension. They should be targeted and modified with three or more antihypertensive drugs used [1,3].

3. In resistant hypertension patients, volume expansion is the most frequently seen pathologic finding. In more than 60% of patients, altering the diuretic therapy either by changing the drug, the dose, or the drug class reduced the prevalence of this finding. It has been shown that switching from the same dose of hydrochlorothiazide to chlorthalidone was associated with an additional drop of 8 mmHg in systolic BP, which leads to more patients achieving their goal BP [1,3].

4. The currently recommended combination of medication is a renin-angiotensin-aldosterone system (RAAS) blocker with a calcium channel blocker (CCB) and a diuretic [1,3].

5. If the BP goal is still not achieved, beta-blockers should be chosen if the pulse rate is more than 60 beats per minute [1,3].

6. Peripheral alpha-blockers are well-tolerated and can be used if beta-blockers do not have alpha-blocking activity. Combining angiotensin-converting enzyme (ACE) inhibitors and angiotensin II receptor blockers (ARBs) has been shown to be less effective in BP reduction. Thus, the drug combination of ACE inhibitors and ARBs are not recommended for resistant hypertension [1,3].

7. Studies suggested that using spironolactone or eplerenone with the existing three-drug treatment in resistant hypertension patients who are obese or have sleep apnea syndrome provides significant BP reduction as seen in Anglo-Scandinavian Cardiac Outcomes Trial (ASCOT), which showed a BP drop of 21.9/9.5 mmHg that was not affected by age, sex, smoking, and diabetic status [17]. The Prevention and Treatment of Hypertension with Algorithm-based Therapy Trial Two (PATHWAY-2) determined that spironolactone was the most effective blood pressure-lowering therapy as compared to bisoprolol or doxazosin. This further suggests that the predominant underlying cause of resistant hypertension is sodium retention, even in patients who are on baseline diuretic therapy. This establishes, for the first time, a clear hierarchy for the drug treatment of resistant hypertension and may revolutionize future treatment guidelines and clinical practice globally [18].

8. If the goal BP is still not achieved with a combination of four drugs then the use of other agents, such as centrally acting alpha agonists (methyldopa/clonidine) or vasodilators (hydralazine/minoxidil) will be needed [1,3].

9. Endothelin receptors antagonists are not currently recommended. However, darusentan, a selective endothelin receptor antagonist (ERA) demonstrated a dose-dependent decrease in the BP of resistant hypertension patients [1,3].

2017 ACC/AHA task force guidelines for resistant hypertension

There are seven steps for the management of resistant hypertension according to the 2017 AHA/ACC. The steps range from the confirmation of the diagnosis to definitive treatment.

1. The resistance needs to be confirmed with measured systolic blood pressure (SBP) ≥130 mmHg and diastolic blood pressure (DBP) ≥80 mmHg along with the patient being on three different antihypertensive drugs at their optimal dose (including one diuretic). An alternative would be a confirmed systolic blood pressure (SBP)/diastolic blood pressure (DBP) <130/80 mmHg and a patient taking more than four antihypertensive medications [3].

2. Pseudo resistance in patients needs to be excluded by double-checking the patients' BP. This would be done during the patient’s outpatient visit. In order to rule out white coat hypertension, BP readings could be done at the patient’s home and work as well. The patient should also be evaluated for nonadherence to antihypertensive medication therapy [3].

3. Identify and reverse lifestyle factors that contribute to resistant hypertension [3].

4. The physician will need to stop or lessen the patient’s use of interfering substances (that contribute to resistant hypertension). These will include NSAIDs, sympathomimetic drugs, stimulants, oral contraceptives, licorice, and ephedra [3].

5. Screen the patient for secondary causes of hypertension. Potential causes would include primary aldosteronism, chronic kidney disease, renal artery stenosis, pheochromocytoma, and obstructive sleep apnea [3].

6. Start pharmacological therapy. The patient is placed on optimized diuretic therapy, mineralocorticoid receptor antagonist, loop diuretics (if he or she has chronic kidney disease and/or is on vasodilators), and other medications with different mechanisms of action [3].

7. Refer the patient to a specialist if the patient’s goal BP has still not been achieved even after six months of consistent therapy. Another reason would be to treat or find a potential cause of secondary hypertension [3].

JNC 8 guidelines over JNC 7 guidelines for patients with resistant hypertension

Studies have demonstrated the failure to achieve target blood pressure in most hypertension patients based on the more stringent JNC 7 guidelines, especially among patients with diabetes and chronic kidney disease. JNC 7 can be envisioned as a set of more idealistic expert recommendations whereas JNC 8 has guidelines based on evidence and the practicality of hypertension management. JNC 8 came in 2014 with lots of controversial issues, as it was a big change from the JNC 7 guidelines. The following changes were seen.

1. JNC 8 raised the target BP to 140/90 in all patients, irrespective of the presence of diabetes mellitus and chronic kidney disease [1,4].

2.  Diuretics were not the first drug of choice in non-resistant hypertension, but they are a definite part of the treatment regimen for resistant hypertension [1,4].

3. Chlorthalidone or indapamide are considered superior therapies over hydrochlorothiazide due to better evidence of benefit [1,4]. 

4. African Americans have a high risk for strokes. Calcium channel blockers (CCBs) have better risk and blood pressure reduction in African Americans vs ACE inhibitors [1,4].

5. Thiazides produce better cardiovascular outcomes (including reduced stroke risk) than ACE inhibitors in African Americans who are “salt-sensitive” [1,4].

6. With the JNC 8 guidelines, patients older than 60 years had target blood pressures of 150/90, making it more lenient for the elderly patients [1,4].

American society for hypertension guidelines for resistant hypertension

The American Society for Hypertension (ASH) guidelines for patients with resistant hypertension have different but validated guidelines for antihypertensives.

1. Patients <80 years of age should start pharmacotherapy at 140/90 mmHg and patients $\geq$80 years are advocated for pharmacological treatment at 150/90 mmHg [19].

2. As advised by JNC 8, patients with diabetes or chronic kidney disease should have a target blood pressure of <140/90 mmHg [19].

3. For patients 18-55 years of age, it was advised to consider lower BP target (e.g., <130/80 mm Hg), per prescriber discretion, if treatment is tolerated. However, evidence of additional benefit with a lower BP target versus BP goal of <140/90 mmHg is lacking [19].

4. It was recommended to blood pressure <130/80 mm Hg in patients with chronic kidney disease with albuminuria [19].

Systolic blood pressure intervention trial study

The Systolic Blood Pressure Intervention Trial (SPRINT) study examined the effects of more intensive antihypertensive therapy than currently recommended in United States adults of age over 50 years who have hypertension and the additional risk for cardiovascular disease [20]. After the median follow-up of 3.26 years, they determined the incidence of primary major adverse cardiac events (MACE) outcome (composite of cardiovascular events) as 25% lower in intensive therapy as compared to the standard group and all-cause mortality reduced by 27% [20]. The “number needed to treat” to prevent primary outcome event or death of 61 and 90, respectively, is also promising. This proves to allow for stricter control of hypertension for middle-aged and elderly patients with cardiovascular disease risk and resistant hypertension [20].

Role of interventions in the treatment of resistant hypertension

Device Therapy

Recently, a novel implantable device (Rheos Systems, CVRx Inc., Minnesota, US) has been developed, which works by electrical stimulation of the carotid sinus. The device enhances afferent nerve traffic from the baroreceptors to the cardiovascular control centers in the brain that reduce the sympathetic outflow and BP. The device consists of a pulse generator and a two-lead system, which are tunneled subcutaneously to each carotid sinus. This is then used as either a unilateral or a bilateral stimulation system. This device provides baroreflex activation based on an algorithm or response of the patient.

In one of the earliest large scale trials, the Rheos Pivotal Trial, there was a mean reduction of 35 mmHg in SBP seen 12 months after baroreflex activation therapy (BAT) [13]. Additionally, 50% of patients had achieved SBP<140 mmHg with BAT therapy. This group also had a 40% reduction of serious adverse side effects for hypertensive urgency [13].

In one study by Scheffers et al., 45 patients with BP more than 160/90 despite three antihypertensive drugs were enrolled and subjected to Rheos Therapy and followed up for a period of two years [14]. After three months, the mean BP was reduced by 21/12 mmHg and the result was sustained in 17 subjects. After two years of follow-up, the mean BP reduction was 33/22 mmHg. The device showed a favorable safety profile.

Renal Artery Angioplasty/Stenting in Patients with Renal Artery Stenosis

Most cases of renal artery stenosis are asymptomatic. Deterioration in renal function may develop if both kidneys are poorly supplied or when treatment with ACE inhibitors is initiated. Some patients present with episodes of flash pulmonary edema.

Atherosclerotic Renal Artery Stenosis

This is initially treated with optimal medical management, including statins, antiplatelet agents, and drugs for the control of blood pressure. When high-grade renal artery stenosis is demonstrated by renal angiography and blood pressure cannot be controlled with medications, or if renal function deteriorates, invasive procedures (angioplasty with or without stenting) can be considered. A 2003 meta-analysis found that angioplasty was safe and effective; however, randomized, controlled trials have not shown any clinical benefit to improve blood pressure or renal function [15].

Fibromuscular Dysplasia

Angioplasty with or without stenting is the best treatment of renal artery stenosis due to fibromuscular dysplasia. 

Sympathetic Denervation by Radio Frequency Ablation

Sympathetic overactivity is thought to be a major contributor to the pathogenesis and progression of hypertension [16,21]. Renal sympathetic activation results in renal vasoconstriction, increased renin secretion, and enhanced sodium and water reabsorption, all of which contribute to the development of hypertension [22].

Renal sympathetic nerves contribute to the development and perpetuation of hypertension, and sympathetic outflow to the kidneys is activated in patients with hypertension. Efferent sympathetic outflow stimulates renin release, increases tubular sodium reabsorption, and reduces renal blood flow. Afferent signals from the kidneys modulate central sympathetic outflow and thereby directly contribute to neurogenic hypertension.

Catheter technology utilizing radiofrequency energy enables selective denervation of the kidney inside the wall of renal arteries [23]. The first study in humans showed successful renal denervation with reduction of sympathetic activity and renin release in parallel with a reduction in central sympathetic outflow. With the advent of a catheter-based technique using radiofrequency to destroy the renal nerves has revitalized the long-abandoned thought of treating hypertension with renal denervation. Following encouraging results of an uncontrolled feasibility trial and a case report, a randomized controlled trial (simplicity hypertension (HTN)-2 (Renal Denervation in Patients with Uncontrolled Hypertension)) demonstrated the potential role of catheter-based renal denervation in the treatment of drug-resistant hypertension [23-26]. Both renal afferent and efferent sympathetic nerves that lie in the wall or adjacent to the wall are crucial to the start and maintenance of systemic hypertension [27-29].

Due to the occasional inadequacy of medical treatment for resistant hypertension, more non-drug treatments like radiofrequency ablation have been created. As a result, a few trials have been conducted to demonstrate the utility of radiofrequency ablation in resistant hypertension patients. One of the earliest studies done was a study by Krum and Schlaich using radiofrequency ablation in 50 patients [24]. In this procedure, a catheter was introduced in each renal artery by the femoral route. Discrete radiofrequency lasting up to two minutes each and of 8 watts or less to obtain up to six ablations separated in each renal artery were given. During ablation, the catheter system monitored tip temperature and impedance according to a predetermined algorithm. Renal angiograms were done at 14-30 days in some patients, and a follow-up angiogram was done in all patients six months after the procedure. The patients had a mean six months office blood pressure reduction of 22/12 mmHg [24].

In the Simplicity-2 trial, Murray de Esler conducted a study using 106 patients. Patients were randomly assigned to undergo renal denervation [26]. The femoral artery was accessed with a standard endovascular ablation catheter, which was advanced into the renal artery and then connected to the radiofrequency generator. Four to six discrete low power radiofrequency treatments were applied along the length of both the main renal arteries. Participants were given conventional heparin and an activated clotting time >250 seconds was maintained [26]. Patients had intra-procedural visceral pain, which was restricted to the duration of delivery of radiofrequency energy and was managed with anxiolytics and narcotics. The results revealed a blood pressure reduction of 31/12 mmHg in patients who underwent sympathetic denervation in SBP and DBP, respectively, as compared to 0/-1 mmHg in patients on medical therapy only [26].

Impressed with the results of both Simplicity-1 and Simplicity-2, a prospective, single-blind, randomized, sham-controlled trial called Simplicity-3 was created [30]. A total of 535 patients with severe resistant hypertension were randomly assigned in a 2:1 ratio to undergo either a renal denervation procedure or a sham procedure. The (mean±SD) change in systolic blood pressure at six months was −14.13±23.93 mmHg in the denervation group as compared with −11.74±25.94 mmHg in the sham-procedure group, for a difference of −2.39 mmHg (95% confidence interval (CI), −6.89 to 2.12; P = 0.26 for superiority with a margin of 5 mmHg). The change in 24-hour ambulatory systolic blood pressure was −6.75±15.11 mmHg in the denervation group and −4.79±17.25 mmHg in the sham-procedure group, for a difference of −1.96 mmHg (95% CI, −4.97 to 1.06; P = 0.98 for superiority with a margin of 2 mmHg). There were no significant differences in safety between the two groups. This trial did not show a significant reduction of SBP in patients with resistant hypertension six months after renal-artery denervation as compared with a sham control [24,30].

Surgical Treatment

Non-selective surgical sympathectomy was effectively used as a treatment of severe hypertension before antihypertensive drugs became easily available [31-34]. Additionally, historic surgical approaches for the treatment of hypertension with renal sympathectomy have long been abandoned due to high perioperative morbidity and mortality [31,33]. During laparotomy, resection of the sympathetic ganglion was done to achieve the effect, but this had a number of side effects, including impotence and urinary and bowel control, and hence, it is discouraged in the present scenario.

## Conclusions

Both JNC 8 and ASH have recommended target goals that are evidence-based, practical, and achievable, despite some variations in guidelines between the two. Although the number of patients who cannot achieve BP goals on multiple drug combinations according to guidelines is growing, resistant hypertension can and should be stringently treated with appropriate and maximum drug therapy after excluding the factors associated with pseudo-resistant hypertension. Unfortunately, so far, there has been no proven benefit of device therapy and radiofrequency ablation techniques in the major trials, and further studies are required in these areas. Although guidelines and recommendations are adequate to meet the requirements of most patients, these are not a substitute for clinical judgment. A multidisciplinary, patient-centered approach is the optimal solution for adequate blood pressure control in the present world.
